# Associations of reported bruxism with insomnia and insufficient sleep symptoms among media personnel with or without irregular shift work

**DOI:** 10.1186/1746-160X-4-4

**Published:** 2008-02-28

**Authors:** Kristiina Ahlberg, Antti Jahkola, Aslak Savolainen, Mauno Könönen, Markku Partinen, Christer Hublin, Juha Sinisalo, Harri Lindholm, Seppo Sarna, Jari Ahlberg

**Affiliations:** 1 Institute of Dentistry, University of Helsinki, Helsinki, Finland; 2 Finnish Institute of Occupational Health, Helsinki, Finland; 3 Finnish Broadcasting Company, Helsinki, Finland; 4 Helsinki University Central Hospital, Helsinki, Finland; 5 Rinnekoti Research Centre, Espoo, Finland; 6 Department of Public Health, University of Helsinki, Helsinki, Finland

## Abstract

**Background:**

The aims were to investigate the prevalence of perceived sleep quality and insufficient sleep complaints, and to analyze whether self-reported bruxism was associated with perceptions of sleep, and awake consequences of disturbed sleep, while controlling confounding factors relative to poor sleep.

**Methods:**

A standardized questionnaire was mailed to all employees of the Finnish Broadcasting Company with irregular shift work (n = 750) and to an equal number of randomly selected controls in the same company with regular eight-hour daytime work.

**Results:**

The response rate in the irregular shift work group was 82.3% (56.6% men) and in the regular daytime work group 34.3% (46.7% men). Self-reported bruxism occurred frequently (often or continually) in 10.6% of all subjects. Altogether 16.8% reported difficulties initiating sleep (DIS), 43.6% disrupted sleep (DS), and 10.3% early morning awakenings (EMA). The corresponding figures for non-restorative sleep (NRS), tiredness, and sleep deprivation (SLD) were 36.2%, 26.1%, and 23.7%, respectively. According to logistic regression, female gender was a significant independent factor for all insomnia symptoms, and older age for DS and EMA. Frequent bruxism was significantly associated with DIS (p = 0.019) and DS (p = 0.021). Dissatisfaction with current work shift schedule and frequent bruxism were both significant independent factors for all variables describing insufficient sleep consequences.

**Conclusion:**

Self-reported bruxism may indicate sleep problems and their adherent awake consequences in non-patient populations.

## Background

According to recent epidemiological data, Finland has a unique pattern of insomnia compared with many other European countries [1]. The high prevalence of insomnia complaints in the Nordic countries has been explained by the dark period during midwinter, which is thought to influence human circadian rhythms.

Shift work has also been shown to affect the circadian rhythm and to be connected with work related problems [2,3]. Moreover, irregular shift work has been implicated as a cause of sleep disorders and tiredness, and may even expose employees to work hazards [4,5]. Currently in the Finnish media industry the production and delivery of radio and TV programmes is in transition from analogue to digital techniques. Technological changes call for new professions and competence requirements, whereas some existing skills are becoming redundant. The 24-hour culture in modern media work, with its irregular shifts and night work, may enhance the psychological pressures of work in an already demanding work environment.

Bruxism has been defined as diurnal or nocturnal parafunctional jaw muscle activity that includes clenching, bracing, gnashing and grinding of teeth [6]. In clinical studies the prevalence of bruxism varies greatly, between 6.5% and 88%, while figures in epidemiologic studies are usually lower, about 6–8% [7-11]. Recent research has increasingly focused on the unsolved etiology of bruxism, and at present, the parafunction is more often thought to be regulated centrally, not peripherally [12]. Evidence also exists that bruxism appears concomitantly with the transient arousal response, and thus may be a sign of a sleep disorder [13,14].

Self-reported bruxism was recently shown among a non-patient population to have a coherent relationship with stress and stress-related disorders [15], and possibly to reflect intrapersonal or interpersonal reactivity [15], or dissatisfaction [16]. It was also found that disrupted sleep associates with bruxism and orofacial pain [17], suggesting a vicious circle between those items. However, whilst clinically detected bruxism may be considered as a sleep disorder in itself, the associations of self-reported bruxism and symptoms and consequenses of disturbed sleep remains far from clear. The aims of the present study, performed in media personnel with or without irregular shift work, were firstly to investigate the occurrence of insomnia symptoms and perceived consequences of insufficient sleep, and secondly to analyze whether self-reported bruxism was associated with them. The effects of some possible confounding factors (viz., restless legs syndrome, snoring, gender, age, and dissatisfaction) relative to sleep quality were controlled.

## Methods

In 2003, a standardized questionnaire was mailed to all employees of the Finnish Broadcasting Company with irregular shift work (n = 750; 57.0% men) and to an equal number of randomly selected controls in the same company with regular eight-hour daytime work (42.4% men). The mean age of invited subjects was 43.0 (SD 10.4) years in irregular shift work and 44.8 (SD 10.2) years in day work. The work duties of the present media personnel included journalism, broadcasting, programme production, technical support and administration.

The overall response rate was 58.3% (53.7% men). The response rate in the irregular shift work group was 82.3% (56.6% men) and in the regular daytime work group 34.3% (46.7% men). The mean age of males in shift work was 45.0 (SD 10.6) years and of females 42.6 (SD 10.7) years (p < 0.001); the corresponding figures for daytime workers were 47.4 (SD 9.7) and 45.5 (SD 10.1) years (NS), respectively [16].

The questionnaire covered demographic items, employment details, general health experience, physical status, insomnia symptoms, psychosocial status, stress, work satisfaction and performance. For the present study, the questionnaire data used were categorized as follows:

a) Demographic data: gender, age.

b) Bruxism: self-assessed frequency of tooth clenching or grinding (never, seldom, sometimes, often, continually) [15-17]. Bruxism was considered as frequent when it occurred 'often' or 'continually'.

c) Dissatisfaction with current workshift schedule (irregular shifts vs. regular day time work)

d) Insomnia symptoms [18,19]: difficulties initiating sleep (DIS), disrupted sleep (DS), and early morning awakenings (EMA). A symptom was considered as present when it occurred at least three nights per week. EMA in the irregular work group means that subjects with the symptom woke up before they intended, despite the hour, and had difficulties in going back to sleep.

e) Perceived consequences of sleep: non-restorative sleep (NRS) (sustained > 1 month), tiredness (at least 3 days per week), sleep deprivation (SLD) (subjective need for sleep 1 h > actual sleep time) [20].

f) Neurological and physical confounding factors affecting sleep quality: restless legs syndrome (RLS): presence of the four essential diagnostic criteria according to the NIH diagnosis and epidemiology workshop for RLS [21], snoring: as perceived or reported by bed partner (at least 3 nights per week)

### Statistical methods

Student's t-test was used to compare continuous variables. The χ^2 ^test was used to study associations between categorical variables. Logistic regression models were fitted to analyse the independent effects of the background variables on the probability of insomnia symptoms (DIS, DS, EMA) and insufficient sleep consequences (NRS, tiredness, SLD). Independent variables included in the six multivariate models were: gender (male = 0, female = 1), age (< 45 yr = 0, ≥ 45 yr = 1), irregular shift work (no = 0, yes = 1), perceived dissatisfaction with current workshift schedule (no = 0, yes = 1), frequent bruxism (often or continually) (no = 0, yes = 1), diagnosed RLS (no = 0, yes = 1), and snoring (at least 3 nights per week) (no = 0, yes = 1). The forced entry method was used, i.e. all selected independent variables were entered in a single step in each regression model. Both dependent and independent variables are described in Table 1.

**Table 1:** Overall percentages of perceived insomnia symptoms and insufficient sleep (1st row) and independent variables (1st column) used in the multivariate models, and occurrences of the studied symptoms by the subgroups. Chi square test.

		**Insomnia symptoms**						**Insufficient sleep**					
	Total n = 874	**DIS **n = 147	P =	**DS **n = 381	P =	**EMA **n = 90	P =	**NRS **n = 316	P =	**Tiredness **n = 228	P =	**SLD **n = 207	P =

Total	%	16,8		43,6		10,3		36,2		26,1		23,7	
Gender:			0,001		0,035		0,012		0,002		0,007		0,077
male	53,7	13,0		40,3		7,9		31,6		22,4		21,3	
female	46,3	21,1		47,4		13,1		41,5		30,4		26,4	
Age:			0,308		0,023		0,036		0,001		0,010		<0,001
< 45	58,1	17,9		40,4		8,5		40,6		29,3		28,7	
≥ 45	41,9	15,3		48,1		12,8		30,1		21,6		16,7	
Irregular shift work:			0,659		0,546		0,388		0,285		0,062		0,843
no	29,4	16,0		42,0		11,7		33,5		21,8		24,1	
yes	70,6	17,2		44,2		9,7		37,3		27,9		23,5	
Dissatisfied with shifts:			0,001		0,091		0,003		<0,001		<0,001		<0,001
no	80,8	14,7		42,2		8,8		33,3		22,5		21,1	
yes	19,2	25,6		49,4		16,7		48,2		41,1		34,5	
Frequent bruxism:			0,001		0,006		0,560		0,006		<0,001		<0,001
no	89,4	15,2		42,7		10,5		34,9		24,6		16,1	
yes	10,6	29,5		58,0		12,5		52,3		46,6		35,2	
RLS:			0,001		0,024		0,002		0,170		0,035		0,354
no	90,6	15,6		42,5		9,3		35,5		25,2		23,3	
yes	9,4	31,0		56,3		21,1		43,7		36,6		28,2	
Snoring:			0,149		0,004		0,853		0,551		0,215		0,320
no	74,1	15,7		40,7		10,2		36,7		25,0		22,8	
yes	25,9	19,9		51,8		10,6		34,5		29,2		26,1	

DIS = difficulties initiating sleep, DS = disrupted sleep, EMA = early morning awakening, NRS = non-restorative sleep, SLD = sleep deprivation, RLS = restless legs syndrome

## Results

Self-reported bruxism occurred frequently in 10.6% of all subjects. The bruxism scores were evenly distributed in the irregular shift work and regular day work groups (NS). A total of 43.6% reported disrupted sleep and 36.2% perceived their sleep non-restorative. The prevalence figures for perceived insomnia and insufficent sleep symptoms and their occurrences by studied subgroups are shown in Table 1.

According to logistic regression models I-III (Table 2), female gender was a significant independent factor for all insomnia symptoms, and older age for DS and EMA. Frequent bruxism was significantly associated with DIS (p = 0.019) and DS (p = 0.021), whilst dissatisfaction with own work shifts was significantly associated with DIS (p = 0.006) and EMA (p = 0.001). RLS was significantly associated with DIS (p = 0.023), as also was snoring with DS (p = 0.010).

**Table 2:** Probabilities of insomnia symptoms by studied independent variables. Logistic regression.

n = 874	**Difficulties initiating sleep **(model I)			**Disrupted sleep **(model II)			**Early morning awakenings **(model III)		
	
	OR	95% CI	P =	OR	95% CI	P =	OR	95% CI	P =
Gender (female)	1,7	1,2–2,6	0,006	1,4	1,0–1,8	0,031	1,7	1,1–2,8	0,020
Age ≥ 45 years	0,8	0,6–1,2	0,313	1,5	1,1–2,0	0,011	1,9	1,2–3,1	0,007
Irregular shift work	0,9	0,6–1,4	0,714	1,1	0,8–1,5	0,690	0,7	0,4–1,1	0,136
Dissatisfied with work shifts	1,9	1,2–2,9	0,006	1,3	0,9–1,9	0,142	2,6	1,5–4,3	0,001
Frequent bruxism	1,9	1,1–3,1	0,019	1,7	1,1–2,7	0,021	1,1	0,6–2,3	0,727
RLS	1,9	1,1–3,2	0,023	1,3	0,8–2,1	0,257	0,8	0,4–1,8	0,655
Snoring	1,4	0,9–2,2	0,125	1,5	1,1–2,1	0,010	1,1	0,6–1,8	0,865

Logistic regression models IV-VI (Table 3) revealed that dissatisfaction with current work shift schedule and frequent bruxism were both significant independent factors for all variables describing insufficient sleep consequences. Female gender was significantly associated with NRS (p = 0.044) and tiredness (p = 0.019). Younger age was significantly associated with NRS (p = 0.009) and SLD (p < 0.001), and snoring with SLD (p = 0.044).

**Table 3:** Probabilities of non-restorative sleep, tiredness and sleep deprivation by studied independent variables. Logistic regression.

n = 874	**Non-restorative sleep **(model IV)			**Tiredness **(model V)			**Sleep deprivation **(model VI)		
	
	OR	95% CI	P =	OR	95% CI	P =	OR	95% CI	P =
Gender (female)	1,4	1,0–1,8	0,044	1,5	1,1–2,1	0,019	1,3	0,9–1,8	0,174
Age ≥ 45 years	0,7	0,5–0,9	0,009	0,8	0,5–1,1	0,108	0,5	0,3–0,7	<0,001
Irregular shift work	1,0	0,7–1,4	0,950	1,1	0,8–1,6	0,587	0,8	0,6–1,2	0,296
Dissatisfied with work shifts	1,7	1,2–2,5	0,005	2,2	1,5–3,2	<0,001	1,8	1,2–2,7	0,005
Frequent bruxism	1,7	1,1–2,7	0,021	2,2	1,4–3,5	0,001	1,8	1,1–2,9	0,022
RLS	1,5	0,9–2,5	0,087	1,2	0,7–2,0	0,461	1,1	0,6–1,8	0,857
Snoring	1,0	0,7–1,4	0,988	1,4	0,9–2,0	0,081	1,4	1,0–2,2	0,044

## Discussion

The present study was performed on media personnel who could be considered as under sustained pressure at work due to intense on-going technological and organizational changes. The study formed part of a comprehensive investigation on shift work and its sleep/awake consequences, and it focused on irregular shift work, which, however, did not emerge as a significant factor in itself. This was a finding that accords with results from earlier studies derived from the present data base [15-17].

Unfortunately, despite several postal reminders, we resulted in a low response rate in the regular day work group. This was partly expected as the study was transparently targeted to examine the health effects of irregular shift work. The invited subjects and respondents in both shift work and day work groups were similar as regards gender and age, which, on the other hand, may modestly suggest that also the day work group could be representative. Nevertheless, due to the uneven response rates the present study may have failed in detecting the actual differences between these two groups.

However, we studied the associations of self-reported bruxism with perceived insomnia symptoms and insufficient sleep using multivariate models in which some confounding factors (viz., restless legs syndrome, snoring, gender, age, and dissatisfaction) relative to sleep quality were simultaneously controlled. Bearing in mind the lower response rate in the day work group, the models were also tested excluding the work group variable, which did not markedly change the effects of the other independent variables. Thus, the work group variable was not considered to be a confounding factor in the models, and further, it was eventually included in the present analyses not to reduce the statistical power.

As the major interest was in self-reported bruxism, the main findings were that frequent bruxism was significantly associated with perceived insomnia symptoms (except EMA) and insufficient sleep. These associations also held in the multivariate analyses. The results may imply a stressful work environment or work dissatisfaction, as discussed earlier [15-17]. The statistically non-significant relationship found between bruxism and EMA, the latter often reportedly associated with depressive mood [18], has also been suggested to be due to the overall low psychological dysfunction found in the present non-patient population [22].

Using questionnaires, as in the present study, may cause difficulties in defining the actual prevalence of bruxism; it may even have been more common among populations but not reported as a behaviour by individuals because of its potential subconscious nature. Or, on the other hand, reporting of bruxism may be influenced by negative affectivity, and individuals with subjective distress may be more likely to perceive, overreact to and complain about their sensations. In the present study bruxism was defined as a subjective perception of tooth grinding or clenching and the definition includes both sleep and awake parafunctions. This also means that sleep and awake bruxism cannot be separated here.

Studies have suggested that stress experience and psychosocial factors may play an important role in the etiology of bruxism [12]. In contrast, evidence also exists that both experienced and anticipated stress associate with awake clenching but would be unrelated to sleep-bruxism recorded with ambulatory devices [23,24]. Polysomnographic studies have revealed, however, that bruxism appears concomitantly with the transient arousal response and has been shown to associate with both sleep quality and sleep architecture [12,14,25]. On the other hand, it is well accepted that stress experiences at work are linked to disturbed sleep and fatigue [26,27]. Thus, if perceived stress or dissatisfaction affect sleep, it could be assumed that they may concomitantly precipitate or amplify bruxism. Further, fatique and pain in the masticatory muscles may be a repercussion of this process.

As regards insomnia symptoms both DIS and DS were found to be markedly more common than previously reported in Finland [1]. On the other hand, the presence of EMA did not differ from that reported in the general population. Also, female gender was overall associated with insomnia symptoms, which is in line with previous epidemiologic findings outside Finland [28-30]. In the present study, age had diverse effects; those ≥ 45 years more often had DS and EMA but yet the younger subjects were more likely to report insufficient sleep complaints. As regards DS this has not been the case in the general population, but it accords with the results found elsewhere. It is noteworthy that DS, also the most significant factor associated with bruxism, emerged as a major sleep disturbance affecting nearly half of subjects in the present non-patient population.

In the multivariate analyses, despite the several associations found cross-sectionally, RLS was significantly associated only with DIS. Snoring, in turn, which was bivariately associated only with DS, was multivariately associated with both DS and SLD. These findings seem logical and they also underscore that neurological or physical factors should be borne in mind when diagnosing and treating insomnia and insufficient sleep problems. Especially in the case of RLS a substantial under-recognition may exist [31-33].

The phenomenon of bruxism may well be genetic in origin, affected psychosocially or pathophysiologically, but is most likely centrally regulated [12]. Yet, despite the increasing number of studies on bruxism, it remains unclear why self-perceived bruxism and polysomnographically or clinically detected bruxism seem to be poorly associated and do not share their etiology. Based on the present study, however, it may be possible to conclude that self-reported bruxism indicates sleep problems and their adherent awake consequences. Also, the found independently detrimental effect of dissatisfaction on sleep should not be ignored.

## Authors' contributions

KA was the main author of the present manuscript and she participated in all stages throughout the work. AJ also took part in planning and writing. AS was the head of the present research project and was vigorously involved in its design and coordination. MK made critical comments on the manuscript and acted as a supervisor. MP and CH were in charge regarding sleep issues whilst JS and HL were consulted as regards cardiovascular and occupational health aspects; they all participated in study planning and writing. SS and JA performed and interpreted the statistical analyses. All authors read and approved the final manuscript.
